# Stem design and sizing influence torsional periprosthetic fracture resistance of cemented stems in osteoporotic femoral models: a comparative biomechanical study

**DOI:** 10.1186/s42836-026-00416-4

**Published:** 2026-07-16

**Authors:** Kohei Hashimoto, Yukio Nakamura, Nobunori Takahashi, Takkan Morishima

**Affiliations:** 1 Department of Orthopedic Surgery, Aichi Medical University, 1-1 Yazakokarimata, Nagakute, Aichi 480-1195 Japan; 2 Division of Osteoporosis, Locomotive Syndrome, Joint Disease Center, Department of Orthopedic Surgery, Aichi Medical University, 1-1 Yazakokarimata, Nagakute, Aichi 480-1195 Japan

**Keywords:** Periprosthetic femoral fracture, Cemented stem, Torsional loading, Biomechanical study, Osteoporotic bone model, Stem sizing, Exeter stem, Charnley–Marcel–Kerboull (CMK) stem, Vancouver classification

## Abstract

**Background:**

Stem sizing may influence periprosthetic femoral fracture (PPF) resistance after cemented total hip arthroplasty (THA), but whether this effect is consistent across cemented stem systems with distinct design philosophies remains incompletely understood. This study investigated how stem sizing influences torsional fracture resistance in an osteoporotic femoral model, using two cemented stem systems—the Exeter polished taper-slip system and the Charnley–Marcel–Kerboull (CMK) polished line-to-line system.

**Methods:**

Four cemented stem constructs were tested using osteoporotic composite femoral analogues (model 3503; Sawbones): Exeter V40 44-3, Exeter V40 44-4, CMK 203, and CMK 303 (*n* = 6 per group). Stem selection was based on CT-derived canal morphology and surgeon judgment of fit, with paired upsizing within the Exeter taper-slip system and paired downsizing within the CMK line-to-line system. Compression–torsion testing was performed under a 2 kN axial load with a 2 N·m internal rotation preload, followed by internal rotation to 40° over 1 s. The primary outcome was fracture torque at failure; secondary outcomes were fracture pattern and Vancouver classification.

**Results:**

Fracture torque differed significantly among constructs (Kruskal–Wallis test, *p* = 0.012). In exploratory, unadjusted pairwise comparisons, the CMK 303—the largest construct—had the highest fracture torque (*p* = 0.013 versus each of the other three constructs); the principal contrast (CMK 303 versus Exeter 44-3) remained significant after Holm correction, whereas the other three constructs did not differ significantly. Median fracture torque was 74.85 N·m (IQR 72.63–81.54), 79.70 N·m (IQR 77.09–82.99), 80.08 N·m (IQR 78.43–82.81), and 94.30 N·m (IQR 86.51–98.90) for the Exeter 44-3, Exeter 44-4, CMK 203, and CMK 303, respectively. These values were 3- to fourfold higher than reported in vivo torsional moments at the hip during routine activity, consistent with an acute traumatic loading condition. Fractures were predominantly Vancouver B2 type (21/24 specimens: all Exeter 44-3 and CMK 203 specimens, four of six Exeter 44-4, and five of six CMK 303).

**Conclusions:**

Stem sizing influenced torsional PPF resistance in this osteoporotic femoral model. The CMK 303, the largest construct tested, demonstrated greater fracture torque than the other three constructs, whereas constructs of similar size showed comparable resistance regardless of stem system. These findings support the concept that the maximum feasible stem size accommodated within a given femur may be a key determinant of acute torsional fracture resistance in cemented osteoporotic constructs, with implications that may extend across cemented stem systems; because the constructs differed in geometry, fixation philosophy, and the manufacturer-recommended cement together, these results are interpreted as construct-level effects rather than as the effect of fixation philosophy alone.

## Background

Total hip arthroplasty (THA) is one of the most successful procedures in orthopaedic surgery, but periprosthetic femoral fracture (PPF) remains one of its most serious complications. Reported incidences after primary THA are low but clinically important because PPF is associated with substantial morbidity, mortality, and healthcare burden [1–3].

Recent registry and meta-analytic data suggest that the risk of postoperative PPF is influenced by femoral stem design and fixation method [4, 5]. Polished collarless tapered stems may produce a characteristic fracture configuration around a well-fixed cemented implant, suggesting that stem geometry and load transfer influence failure behaviour [6]. However, clinical and registry studies cannot isolate the mechanical factors responsible for fracture. In cemented THA, stem behaviour is influenced by multiple construct-related variables, including stem shape, surface finish, subsidence characteristics, and cement–bone interface mechanics [7]. These factors may be particularly relevant in osteoporotic bone, where rotational instability and reduced structural reserve may predispose to torsional failure.

Among construct-related variables, stem sizing has emerged as a potentially important determinant of acute fracture resistance. Larger-bodied cemented stems have been shown to increase torque to failure in biomechanical models, while shorter or undersized cemented stems may reduce periprosthetic fracture resistance [8, 9]. In an osteoporotic composite bone model, Jain et al. examined a polished taper-slip stem and reported that construct-related variables modulated mechanical resistance to postoperative PPF [10]. We have also previously shown that, when stem shape and size are held constant, cemented stems of different alloy compositions produce similar fracture torque and a reproducible Vancouver B2-like fracture pattern in bone models [11], and that stems of different lengths can yield comparable fracture torque when proximal stem volume is similar [12]. Together, these observations raise the possibility that stem sizing, rather than alloy or stem length per se, may be a key determinant of acute torsional fracture resistance.

This question is also relevant across cemented stem systems with distinct design philosophies. The Exeter system represents a classical double-tapered polished taper-slip (force-closed) concept, in which controlled subsidence within a relatively thick cement mantle is integral to fixation. The Charnley–Marcel–Kerboull (CMK) stem represents a quadrangular polished tapered stem implanted with a line-to-line cementing technique that uses a thin cement mantle and direct cortical contact, conceptualised as the “French paradox” [13, 14]. Although both stems share a polished surface and a tapered geometry, they differ markedly in their fixation philosophy, in the cement mantle they generate, and in their relationship to the endosteal cortex. Conceptually, the taper-slip stem subsides slightly within a relatively thick cement mantle, converting axial load into radial (hoop) compressive stress transmitted through the cement to the proximal femur, whereas the line-to-line stem transfers load more directly to the endosteal cortex through a thin cement mantle; the two philosophies therefore generate different proximal cement-mantle and cortical stress distributions. Whether stem sizing similarly influences torsional fracture resistance across these two distinct systems has not been directly examined in osteoporotic bone.

Therefore, we performed a comparative biomechanical study using osteoporotic femoral models to investigate how stem sizing influences torsional periprosthetic fracture resistance, using paired stem sizes within each of the two cemented stem systems described above. Our primary objective was to compare fracture torque among the four constructs. Our secondary objectives were to characterise fracture pattern and to interpret the mechanical implications of these findings for periprosthetic fracture around cemented stems.

## Methods

### Study design

This was a comparative biomechanical study designed to evaluate torsional periprosthetic fracture resistance around cemented femoral stem constructs in osteoporotic femoral models. The study was designed as a controlled in vitro failure test rather than a simulation of in vivo loading; the loading protocol was selected to permit reliable comparison of acute torsional fracture resistance among constructs under standardised conditions, following established biomechanical methodology [8, 9, 15, 16].

### Stem selection and construct rationale

Stem constructs were selected from two cemented stem systems with distinct design philosophies: the Exeter V40 polished taper-slip system and the CMK polished line-to-line (French paradox) system [13, 14]. Both stems share a polished surface and a tapered geometry, but differ in fixation philosophy and in the cement mantle they generate; the Exeter is a double-tapered collarless stem implanted with a relatively thick cement mantle and is designed to subside within the cement mantle by a taper-slip mechanism, whereas the CMK is a quadrangular collared stem implanted with a line-to-line technique and a thin cement mantle, with primary stability conferred by direct contact between the stem and the endosteal cortex.

Within each system, paired stem sizes were tested to specifically isolate the effect of stem sizing in the same osteoporotic femoral morphology. Based on CT-based assessment of canal morphology and surgeon judgment of fit in the femoral model, the Exeter V40 44-3 and the CMK 303 stems were initially chosen as the constructs that best matched their respective design concepts within the same osteoporotic femoral morphology. Because the Exeter system allows insertion of a one-size larger stem using the same rasp, an Exeter V40 44-4 stem was additionally included to reflect clinically relevant upsizing within the same preparation. In contrast, for the CMK system, a one-size smaller stem (CMK 203) was additionally selected to reflect clinically relevant downsizing relative to the initial construct. Accordingly, four stem constructs were tested: Exeter V40 44-3, Exeter V40 44-4, CMK 203, and CMK 303 (Table 1). Implant specifications are summarised in Table 1.

**Table 1:** Implant specifications

Variable	Exeter V40 44-3	Exeter V40 44-4	CMK 203	CMK 303
Design concept	Polished taper-slip	Polished taper-slip	Polished line-to-line (French)	Polished line-to-line (French)
Stem length, mm	150	150	115	125
Stem offset, mm	44	44	43	46.6
Neck-shaft angle, °	125	125	130	130
Stem geometry	Double-tapered	Double-tapered	Quadrangular tapered	Quadrangular tapered
Surface finish	Polished	Polished	Polished	Polished
Stem material	Stainless steel	Stainless steel	Stainless steel	Stainless steel
Collar	Collarless	Collarless	Collared	Collared
Cement type	Simplex P (medium)	Simplex P (medium)	Optipac (high)	Optipac (high)

Implant specifications are based on manufacturer’s information

*CMK* Charnley–Marcel–Kerboull

### Experimental specimens

All constructs were prepared using a left, medium, osteoporotic composite femur analogue (model 3503; Sawbones, Pacific Research Laboratories, Vashon, WA, USA). This model has a thin-walled, low-density cortical shell and 10 pcf cancellous foam (approximately 0.16 g/cm^3^), and is commonly used to simulate osteoporotic bone quality. Six specimens were used for each stem construct.

### Sample size

Sample size was determined a priori on the basis of the loading protocol reported by Morishima et al. [9]. With an expected difference in fracture torque of 40 N·m, a standard deviation of 20 N·m, α = 0.05, and power (1 − β) = 0.80, at least six specimens per group were required. Six replicates were therefore used for each construct.

### Implantation and cementing technique

All stems were implanted according to the design concept and manufacturer-recommended surgical technique for each system. The Exeter constructs were implanted using sequential rasping and a relatively thick cement mantle, in keeping with the taper-slip philosophy. The CMK constructs were implanted with a line-to-line technique using broaches matched to the stem dimensions, in keeping with the French cementing technique [13]. For cementation, a distal cement restrictor was placed in a standardised position immediately distal to the planned stem tip, corresponding to the position routinely used during cemented stem implantation, so that the length of the distal cement column below the stem tip was kept consistent across the four constructs; a distal centraliser appropriate to each stem was also used. To reflect routine clinical practice, the polymethylmethacrylate (PMMA) bone cement recommended for each system was used. Surgical Simplex P PMMA bone cement (medium viscosity; Stryker Orthopaedics, Kalamazoo, MI, USA) was used for the Exeter constructs, whereas Optipac PMMA bone cement (high viscosity; Zimmer Biomet, Warsaw, IN, USA) was used for the CMK constructs. The polymer powder and monomer liquid were vacuum-mixed according to the manufacturer’s instructions for use, and the working/setting time was approximately 10 min at 21 °C.

Both systems used medium- or high-viscosity cement only; low-viscosity cement, which has previously been associated with increased risk of periprosthetic fracture, was not used in any construct. Each stem was inserted at 20° anteversion with neutral varus/valgus alignment, using the central axis of the distal tapered portion as reference. Post-cementation anteroposterior and lateral radiographs confirmed alignment within ± 1° of the intended position. The constructs were prepared by an experienced arthroplasty surgeon (T.M.). All constructs were tested approximately 24 h after cementation to allow for cement curing. The potential influence of residual stresses arising from cement polymerisation and stem insertion was not directly quantified in the present study and is acknowledged as a limitation.

### Mechanical loading test and rationale for loading axis

**Fig. 1:**
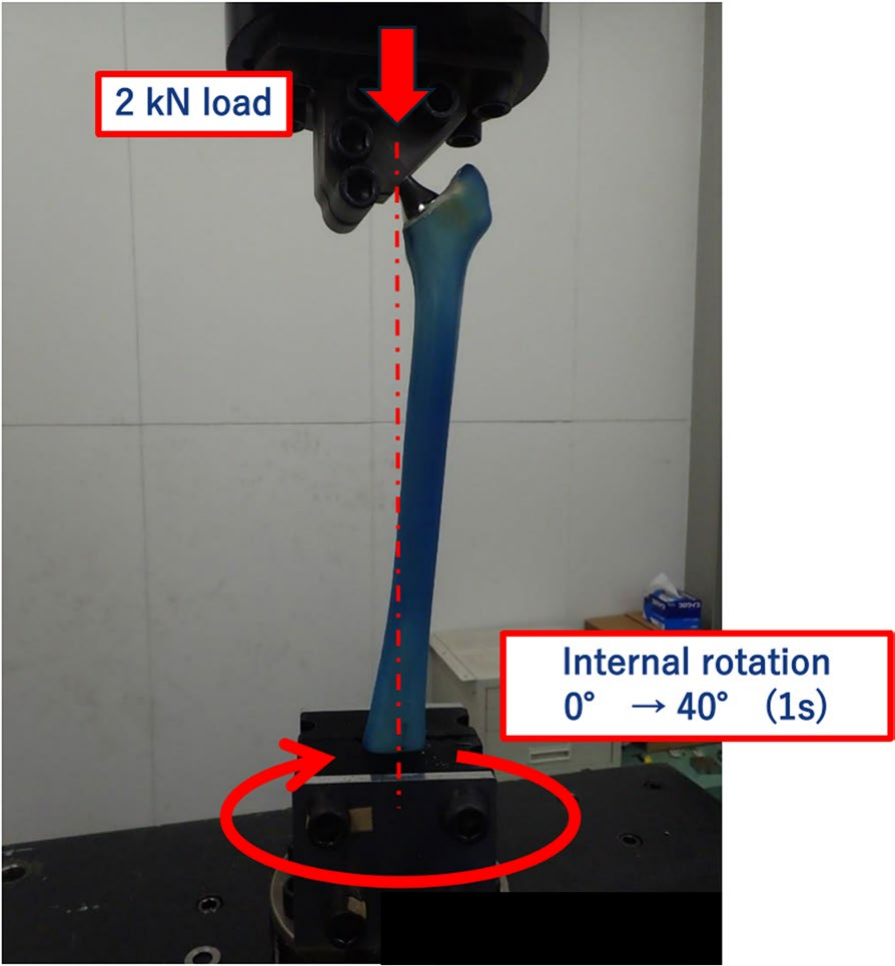
Mechanical loading test. The proximal femur is attached at the centre of rotation of the implant head by means of a clamp. The femoral head is located in the vertical loading axis of the machine. A 2 kN axial compressive load was applied through the actuator, and the implant was internally rotated to 40° over 1 s about this axis. As described in the Methods, this standardised loading axis was chosen to produce a reproducible torsional failure mode and does not reproduce the in vivo loading axis

Compression–torsion testing was performed using a CMH biaxial material testing system (Saginomiya Seisakusho, Nagoya, Japan), which enables simultaneous application of axial compression and torsion. The proximal femur was clamped at the centre of rotation of the implant head, and the vertical loading axis of the machine was aligned through the centre of the femoral head and the intercondylar notch. The distal femur was rigidly secured in a custom fixture (Fig. 1).

Each construct was tested under combined loading to reproduce fracture patterns in the femoral analogue while allowing comparative evaluation among stem constructs. A 2 kN axial compressive load and an internal rotation preload of 2 N·m were applied and maintained, and the implant was then internally rotated to 40° over 1 s (40°/s) in phase (0° lag) with the axial load, following the methodology of Morishima et al. [9]. The combination of a sustained axial load with rapid internal rotation to 40° within 1 s was selected to approximate the high-rate torsional overload that occurs when the body rotates about a relatively fixed femur, such as during a fall onto the side or a twisting misstep, which is the mechanism clinically associated with acute torsional periprosthetic fracture. We acknowledge that, because the hip is a ball-and-socket joint, the moment that can develop about the femoral head–acetabulum axis in vivo is constrained by joint geometry, and that physiological torsional moments transmitted to the femoral construct typically act through indirect lever arms originating from ground reaction forces and muscle action [17]. The axis used in the present study therefore does not reproduce the in vivo loading axis. Rather, this protocol was selected because it generates a reproducible torsional failure mode at the bone–cement–stem construct that allows controlled comparison among stems, and because it has been adopted as a standardised acute torsional failure test in multiple prior biomechanical studies of cemented PPF [8, 9, 15, 16]. The clinical relevance of this protocol is supported by its tendency to generate Vancouver B2-type fracture patterns that mirror those clinically observed around well-fixed cemented polished tapered stems [6, 15]. The findings should therefore be interpreted as comparative measures of construct torsional resistance under standardised acute failure conditions, not as predictions of absolute in vivo loading thresholds.

### Outcome measures

The primary outcome measure was fracture torque at failure, defined as the peak torque recorded at the moment of catastrophic failure. Secondary outcomes included fracture pattern. Fracture patterns were classified according to the Vancouver system. Two authors independently assessed the fracture patterns, and discrepancies were resolved by consensus.

### Statistical analysis

Statistical analyses were performed using JMP Pro 14 (SAS Institute Inc., Cary, NC, USA) for macOS. Because fracture-torque data were not normally distributed and the sample size per group was small (*n* = 6), non-parametric methods were used. Overall differences among the four stem constructs were assessed using the Kruskal–Wallis test, which served as the primary (confirmatory) inferential test. Given the small sample size, the subsequent pairwise comparisons (Wilcoxon rank-sum test) were regarded as exploratory and are reported as unadjusted, descriptive *p*-values, without correction for multiple comparisons; they were not used as a basis for confirmatory inference. To assess the robustness of the principal contrast, Dunn’s test with Holm correction for multiple comparisons was additionally applied. Statistical significance for the Kruskal–Wallis test was set at *p* < 0.05 (two-sided). Box plots display the median, interquartile range, whiskers extending to 1.5 × the interquartile range, and individual data points.

## Results

Fracture torque differed significantly among the four stem constructs (Kruskal–Wallis test, *p* = 0.012). In exploratory, unadjusted pairwise comparisons, the CMK 303 had the highest fracture torque, exceeding the Exeter 44-3, Exeter 44-4, and CMK 203 (*p* = 0.013 for each, Wilcoxon rank-sum test; not corrected for multiplicity and interpreted descriptively), whereas the remaining three constructs did not differ significantly from one another. The principal contrast—CMK 303 versus the smallest construct, Exeter 44-3—remained significant after Dunn’s test with Holm correction (adjusted *p* = 0.013), whereas the contrasts of the CMK 303 with the Exeter 44-4 and CMK 203 did not retain significance after correction, consistent with a graded rather than categorical effect of construct size.

**Fig. 2:**
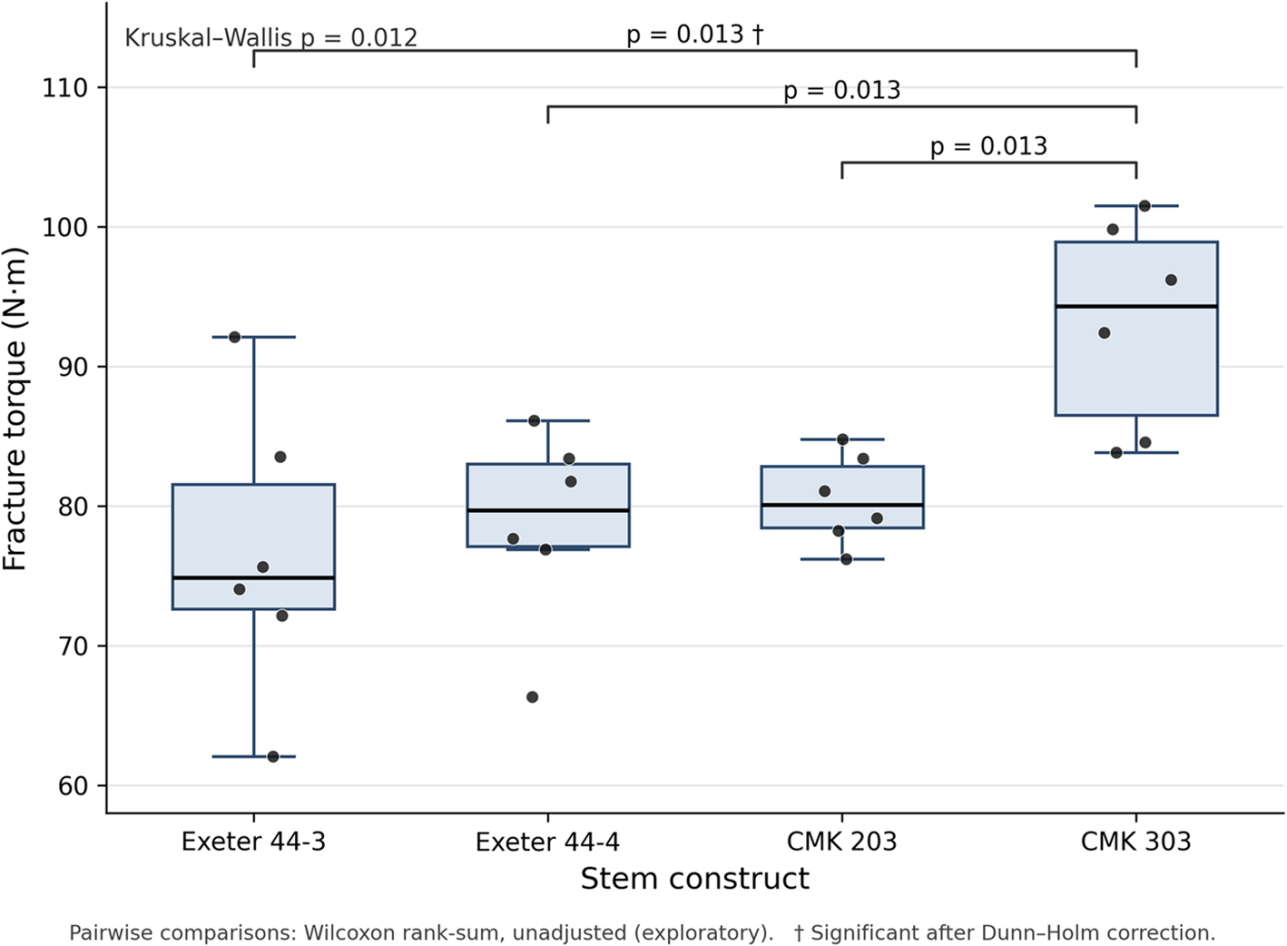
Biomechanical testing results. Box plots comparing fracture torque among four different cemented hip stem constructs: Exeter 44-3, Exeter 44-4, CMK 203, and CMK 303. Each box represents the interquartile range (25th–75th percentile), the horizontal line within each box indicates the median, and the whiskers represent values within 1.5 × the interquartile range. Individual data points are overlaid as black dots. The overall difference among constructs was significant (Kruskal–Wallis *p* = 0.012). Pairwise comparisons (Wilcoxon rank-sum test) are exploratory and unadjusted for multiplicity: the CMK 303 exceeded the Exeter 44-3, Exeter 44-4, and CMK 203 (*p* = 0.013 for each), with no significant differences among the other three constructs. After Dunn’s test with Holm correction, only the CMK 303 versus Exeter 44-3 contrast remained significant (adjusted *p* = 0.013)

Median fracture torque was 74.85 N·m (interquartile range [IQR] 72.63 to 81.54) for the Exeter 44-3, 79.70 N·m (IQR 77.09 to 82.99) for the Exeter 44-4, 80.08 N·m (IQR 78.43 to 82.81) for the CMK 203, and 94.30 N·m (IQR 86.51 to 98.90) for the CMK 303 (Fig. 2).

Fracture patterns were predominantly Vancouver B2 type. Of the 24 specimens, 21 showed a B2-type fracture, and three showed a Vancouver C-type fracture. All Exeter 44-3 and CMK 203 specimens fractured in a B2 pattern. In the Exeter 44-4 group, four specimens showed a B2 fracture, and two showed a C-type fracture. In the CMK 303 group, five specimens showed a B2 fracture, and one showed a C-type fracture (Table 2).

**Table 2:** Fracture torque and fracture pattern by stem construct

Stem construct	*n*	Median fracture torque (IQR), N·m	Vancouver B2, *n*	Vancouver C, *n*
Exeter 44-3	6	74.85 (72.63 to 81.54)	6	0
Exeter 44-4	6	79.70 (77.09 to 82.99)	4	2
CMK 203	6	80.08 (78.43 to 82.81)	6	0
CMK 303	6	94.30 (86.51 to 98.90)	5	1

Data are presented as median (IQR), unless otherwise specified

*IQR* interquartile range

## Discussion

The principal findings of this study were as follows. First, torsional fracture resistance differed among the four cemented stem constructs tested in an osteoporotic femoral model, with the CMK 303 construct—the largest construct in our model—demonstrating significantly greater fracture torque than the Exeter 44-3, Exeter 44-4, and CMK 203. Second, fracture torque did not differ significantly among the Exeter 44-3, Exeter 44-4, and CMK 203, three constructs of broadly comparable size despite belonging to two different cemented stem systems. Third, fracture patterns were predominantly Vancouver B2 type, indicating that combined axial compression and internal rotation produced a reproducible periprosthetic fracture pattern in this osteoporotic model. Taken together, these findings suggest that, within the range tested here, stem sizing exerted a greater influence on acute torsional fracture resistance than the underlying stem fixation philosophy.

### Clinical relevance of measured torque magnitudes

To place the present fracture torques into clinical context, the median values of 74.85–94.30 N·m may be compared with reported in vivo torsional loads at the hip. Telemetric studies of instrumented hip prostheses have reported peak torsional moments of approximately 18–23 N·m about the femoral component during routine activities such as level walking and stair ascent, and these may rise transiently during stumbling [17–19]. The fracture torques observed in the present study are therefore approximately 3- to 4-fold higher than peak physiological torsional moments at the hip during routine activity, consistent with the interpretation that periprosthetic torsional failure of an osteoporotic cemented construct requires a supra-physiological loading event such as a fall onto the side, an unexpected rotational injury, or a misstep. Importantly, the relative differences in fracture torque among constructs (the CMK 303 produced approximately 18–26% higher torque than the other three constructs) are of comparable magnitude to the differences between physiological and supra-physiological loading regimens, suggesting that construct selection may meaningfully shift the threshold at which traumatic loading produces fracture in osteoporotic bone.

### Stem sizing as a key mechanical factor

The pattern of results in this study supports stem sizing as a key determinant of acute torsional fracture resistance in osteoporotic cemented constructs. Three observations support this interpretation. First, only the CMK 303, the largest construct tested in our model, showed significantly greater fracture torque than the other constructs; the remaining three constructs, of broadly comparable size, were not significantly different from one another despite belonging to two different stem systems with distinct fixation philosophies. Second, within the Exeter system, modest upsizing from 44-3 to 44-4 numerically increased fracture torque without reaching statistical significance, consistent with the modest dimensional change between these two paired sizes. Third, within the CMK system, downsizing from 303 to 203 produced a marked numerical decrease in fracture torque, consistent with the more substantial dimensional change. We note that within each stem system, the upsized construct differed from its smaller counterpart in multiple dimensions simultaneously (body size, length, and offset), and these factors cannot be dissociated within the present design; nevertheless, the cumulative dimensional change is what we refer to here as “stem sizing”. Throughout this study, therefore,“stem sizing” denotes construct-level differences associated with implant size selection—a bundle of co-varying features (body dimensions, offset, length, and shoulder/collar geometry) and, between systems, the fixation philosophy and the manufacturer-recommended cement—rather than the isolated effect of any single geometric variable. The findings should therefore be interpreted as comparisons between whole constructs rather than as the isolation of one parameter.

These findings are broadly consistent with previous experimental work showing that stem size and geometry influence torsional fracture resistance. Morishima et al. reported that short cemented stems had lower fracture torque than standard stems, suggesting that reduced stem length may compromise resistance to periprosthetic fracture [9]. Ginsel et al. subsequently showed that larger-bodied cemented femoral components can increase fracture torque, supporting the importance of stem volume and proximal load transfer [8]. In a Sawbone model, Windell et al. demonstrated that torque to failure differed among three polished tapered cemented stems, with the Exeter construct showing greater fracture torque than the CPT and C-Stem [15]. Takegami et al., using a similar experimental model with three cemented stem types, reported that fracture torque and fracture pattern varied by construct, with CPT showing lower fracture torque than CMK [16]. Taken together, these studies and the present results suggest that stem sizing is an important determinant of acute torsional fracture resistance, although the design philosophies of the implants involved differ across studies. To clarify how the present study relates to this body of work, Table 3 summarises the experimental features and principal findings of these prior biomechanical studies alongside the present study. The novelty of the present study lies not in the torsional failure test itself, but in applying a standardised acute torsional failure model to osteoporotic femoral analogues while comparing stem sizing across two cemented systems with distinct fixation philosophies.

**Table 3:** Comparison of prior biomechanical studies of torsional periprosthetic fracture and the present study

Study	Femoral model	Bone quality	Stem system(s)	Size/design comparison	Loading protocol	Principal finding
Morishima 2014 [9]	Composite Sawbone (model 3403)	Standard	Cemented Exeter V40 (125 vs 150 mm)	Stem length	2 kN axial + IR to 40°/1 s	Standard-length > short (e.g., 44 offset 156.2 vs 131.7 N·m); Vancouver B2
Ginsel 2015 [8]	Composite Sawbone	Standard	Cemented Exeter (larger-bodied)	Stem body size	Biaxial axial + torsion	Larger body increased torque (Exeter 44-4 ≈ 237.1 N·m)
Windell 2021 [15]	Sawbone	Standard	Exeter, CPT, C-Stem (polished tapered)	Stem design	Axial + torsion	Reproducible Vancouver B2; Exeter highest (≈ 174 N·m) > CPT, C-Stem
Takegami 2022 [16]	Composite femur	Standard	Three cemented stems (incl. CMK, CPT)	Stem design	Axial + torsion	Varied by construct; CMK 203 ≈ 200.5 N·m; CPT < CMK
Jain 2024 [10]	Composite femur (model 3503)	Osteoporotic	Cemented polished taper-slip (Exeter 37.5-1)	Construct variables	Standardised torsional loading	Construct variables modulated PPF resistance
Hashimoto 2025 [11]	Composite Sawbone (model 3403)	Standard	Same-sized cemented VLIAN (Co–Cr–Mo vs SUS)	Stem material	2 kN axial + IR to 40°/1 s	No difference by material (103.0 vs 98.7 N·m, *p* = 0.575); Vancouver B2
Hashimoto 2025 [12]	Composite femur (model 3503)	Osteoporotic	AMIS-K vs CMK 303	Stem length (similar proximal volume)	2 kN axial + IR to 40°/1 s	Comparable torque despite different stem lengths
Present study	Composite femur (model 3503)	Osteoporotic	Exeter taper-slip + CMK line-to-line	Paired sizing within each system	2 kN axial + IR to 40°/1 s	CMK 303 (largest) highest (94.30 N·m); other three comparable (74.85–80.08 N·m); predominantly B2

Bone quality reflects the composite-femur density grade (model 3403, standard; model 3503, low-density/osteoporotic). Values are as reported in the cited studies; cross-study comparisons should be interpreted with caution because of differences in model, technique, and apparatus

*CMK* Charnley–Marcel–Kerboull, *CPT* collarless polished tapered, *IR* internal rotation, *PPF* periprosthetic femoral fracture, *SUS* stainless steel

The present findings also extend our previous work. In our same-sized material comparison study, fracture torque did not differ significantly between cobalt–chromium and stainless-steel cemented stems when stem size and construct conditions were held constant, suggesting that the mechanical influence of alloy composition may be masked by size-related factors [11]. This prior observation also provides indirect evidence against a dominant role of cement viscosity in determining fracture torque: in that study, construct conditions including cement type were held constant, and no significant difference in torque was detected despite variation in stem alloy. In a separate comparison between AMIS-K and CMK 303, fracture torque was also similar despite differences in stem length, implying that a shorter stem with sufficient proximal volume may preserve fracture resistance [12]. Jain et al. similarly used the same osteoporotic composite femoral model and reported that construct-related variables influenced fracture resistance around a cemented polished taper-slip stem under experimental loading [10]. Notably, the Exeter construct tested in that study was a relatively small 37.5-1 stem, which may have contributed to its comparatively low fracture torque. This interpretation is consistent with the present findings and supports the view that the size of the stem that can ultimately be implanted within a given femur may be a key determinant of fracture resistance.

The present findings may also be placed in broader context by considering the influence of bone quality. Takegami et al. reported a median fracture torque of 200.5 N·m for a CMK 203 stem in a standard-density composite femur analogue [16], compared with 80.08 N·m observed for the same construct in the present osteoporotic series. Ginsel et al. reported 237.1 N·m for an Exeter 44-4 stem in standard-density bone [8], compared with 79.70 N·m in the present study, and Windell et al. reported 174 N·m for the Exeter V40 in a standard-density model [15], compared with 74.85 to 79.70 N·m for the Exeter constructs in the present osteoporotic series. While such cross-study comparisons should be interpreted cautiously because of differences in cementing technique, specimen generation, and testing apparatus, the consistent magnitude of reduction across multiple implant types and independent studies strengthens the inference that osteoporotic bone substantially narrows the mechanical reserve against periprosthetic fracture, and may also limit the absolute benefit conferred by stem upsizing.

### Comparing two cemented stem systems

Although the present study was not designed as a head-to-head comparison of stem fixation philosophies, the inclusion of two cemented stem systems with distinct fixation philosophies—the Exeter polished taper-slip system and the CMK polished line-to-line system—allows some observations on this point. Both systems share a polished surface and a tapered geometry, but differ markedly in their fixation philosophy and in the cement mantle they generate. The Exeter relies on controlled subsidence within a relatively thick cement mantle (taper-slip/force-closed mechanism) [7], whereas the CMK relies on direct cortical contact through a thin cement mantle (the “French paradox” concept) [13]. Despite these conceptual differences, the three constructs of broadly comparable size (Exeter 44-3, Exeter 44-4, CMK 203) produced comparable fracture torque, while the largest construct (CMK 303) clearly outperformed the others. This pattern suggests that, at least in osteoporotic bone under acute torsional loading, stem fixation philosophy alone does not determine fracture resistance; rather, the size of the stem that can be safely accommodated within a given femur may be more influential. Because the two systems also differed in their manufacturer-recommended cement (Simplex P for the Exeter and Optipac for the CMK) and in implant geometry, the observed differences reflect whole-construct effects and should not be attributed to fixation philosophy—or to cement—in isolation.

Numata et al. reported that the CMK stem with a thin cement mantle implanted by the line-to-line technique can achieve mechanically favourable behaviour, despite challenging conventional cementing principles [13]. The present results are not inconsistent with that report; the highest fracture torque in our study was produced by the largest CMK construct, which combines the line-to-line philosophy with the largest physical dimensions among the constructs tested. However, the comparable performance of the smaller CMK 203 with the two Exeter constructs suggests that the mechanical advantage of the CMK design is not automatic, but is realised when stem size is sufficient relative to the femoral canal.

Our finding that a larger construct with a thinner cement mantle (CMK 303) showed the highest torsional resistance may appear to contrast with registry data showing a higher incidence of periprosthetic femoral fracture with uncemented than with cemented stems [3]. The two observations are, however, compatible, because they concern different fixation modes and different fracture mechanisms. The elevated fracture risk of uncemented stems is largely driven by hoop stresses generated during press-fit insertion and by the absence of a load-distributing cement mantle at the implant–bone interface. In a cemented construct, by contrast, the cement mantle distributes load, and a larger stem increases the polar moment of inertia and overall construct stiffness, raising the torque required for acute failure even when the mantle is thin. The protective effect of a larger stem observed here therefore operates within cemented constructs and does not extrapolate to the press-fit failure mechanism of uncemented stems.

### Clinical implications

In osteoporotic bone, maximising the stem size that can be safely accommodated within the femur may be more important than focusing on fixation philosophy, material, or stem length alone. From a practical perspective, these findings may support the concept of gentle rasping or broaching and insertion of the maximum feasible stem size for a given femoral canal. At the same time, excessive enlargement during preparation may increase the risk of intraoperative fracture, particularly in fragile bone. These findings should therefore be interpreted as mechanistic support for careful construct sizing in osteoporotic bone rather than as direct evidence for the superiority of any single implant in clinical practice. In interpreting these results clinically, the way each system is sized is as important as the sizes themselves. For this femoral geometry, the conceptually matched sizes were the Exeter 44-3 and the CMK 303, so each system was tested at the size it would normally receive in this canal; the Exeter 44-4 and CMK 203 were added to represent clinically realistic up- and down-sizing within each system. Because the polished taper-slip Exeter seats by wedging within the cement, a 44-4 stem can be implanted into the preparation made for a 44-3 without additional canal removal, whereas the line-to-line CMK requires the canal to be prepared to the stem size, so that achieving the largest body demands more extensive rasping and a correspondingly higher risk of intra-operative fracture, particularly in osteoporotic bone. The Exeter 44-3 and 44-4 did not differ in fracture torque, indicating that up-sizing the Exeter, and the thinner cement mantle this produces, did not compromise acute torsional resistance in this model, although the longer-term consequences of a thinner mantle for fixation were not assessed here; the Exeter also has an established long-term clinical record. The CMK 303 produced the highest fracture torque, significantly greater than all other constructs, confirming a genuine mechanical advantage of the largest line-to-line construct when it can be properly seated. Importantly, however, this advantage argues for restraint rather than for routinely forcing the largest possible body: the CMK 203 already reached a fracture torque within the range of the clinically successful Exeter constructs, so where seating the larger CMK would require the over-rasping that predisposes to intra-operative fracture, the 203 may achieve clinically adequate torsional resistance without that risk. These interpretations should be read cautiously, because the study was not designed to demonstrate equivalence and rests on a single canal geometry.

### Fracture pattern interpretation

The predominance of Vancouver B2-type fractures in this study is also notable. Windell et al. reported a reproducible Vancouver B2 fracture pattern across all tested polished tapered cemented stems in a Sawbone model [15], and Grammatopoulos et al. described a characteristic periprosthetic fracture configuration around well-fixed polished tapered collarless stems in clinical practice [6]. The present findings are broadly consistent with that literature. Although the Exeter 44-4 and CMK 303 showed occasional Vancouver C-type fractures, the limited number of such cases precludes firm interpretation. Nonetheless, the distribution suggests that differences in construct geometry may influence not only fracture torque but also the eventual fracture path under acute torsional loading. Before testing, we hypothesised that insertion of a larger stem might increase proximal stiffness while accentuating the mechanical mismatch distal to the stem tip, thereby predisposing the construct to a more distal fracture pattern. Consistent with this hypothesis, when the present findings are considered together with our previous experimental results [12], constructs showing C-type fractures tended to exhibit relatively high fracture torque, raising the possibility that increased proximal construct volume may improve overall fracture resistance while at the same time shifting the eventual fracture path more distally.

### Limitations

This study has several limitations. First, all experiments were performed using synthetic osteoporotic femoral analogues rather than cadaveric bone, and therefore the results may not fully reproduce the heterogeneity of human femoral morphology and bone quality and do not reproduce the constraint provided by surrounding soft tissues such as muscle and joint capsule. Second, the sample size was small, although it was based on an a priori power calculation and was sufficient to detect overall group differences in fracture torque. Third, different cement systems were used for the Exeter and CMK constructs to reflect routine manufacturer-recommended clinical practice; while this improves clinical realism, it also introduces a potential confounding factor. Higher-viscosity cement is generally considered to have greater stiffness, and if cement properties contributed to fracture torque, this would introduce a bias favouring higher torque in CMK constructs. However, our previous study in which cement type was held constant demonstrated no significant difference in fracture torque between stems of different alloy compositions [11], suggesting that structural factors related to construct geometry exert a greater influence on torsional resistance than cement composition alone. Fourth, residual stresses arising from cement polymerisation and from frictional forces during stem insertion were not directly quantified. Although all constructs were tested approximately 24 h after cementation under uniform conditions, any systematic difference in residual stress between systems could in principle have contributed to the observed differences. Fifth, the loading model represented an acute standardised compression–torsion failure test about a single rotational axis through the femoral head; this protocol was chosen to permit reproducible comparison among constructs, but it does not reproduce cyclic loading, muscle forces, or the more complex loading geometries that occur in vivo. Sixth, the study evaluated a limited number of stem sizes within each of two cemented stem systems, and therefore the results should be interpreted at the level of the specific constructs tested. Finally, all constructs were implanted in a single standardised femoral analogue with one canal geometry. Within this geometry, both matched sizes, the Exeter 44-3 and the CMK 303, corresponded to the size each system would normally receive, so neither system was tested outside its intended fit; nevertheless, a single canal geometry cannot capture the full range of femoral morphology, and the relative performance of these constructs may differ in narrower or wider canals. The results should therefore be read as construct-level comparisons within one canal geometry rather than as universally applicable rankings.

## Conclusions

Stem sizing influenced torsional periprosthetic fracture resistance in osteoporotic femoral models. The CMK 303, the largest construct tested, demonstrated greater fracture torque than the Exeter 44-3, Exeter 44-4, and CMK 203, whereas the latter three constructs—spanning two cemented stem systems with distinct fixation philosophies but of broadly comparable size—showed comparable resistance. Fracture patterns were predominantly Vancouver B2 type under the present testing conditions. The fracture torques recorded were approximately 3- to 4-fold higher than peak physiological hip torsional moments, consistent with an acute traumatic loading regime. These findings support the concept that the maximum feasible stem size accommodated within a given femur may be a key determinant of acute torsional fracture resistance in cemented osteoporotic constructs. Because the two systems differed not only in fixation philosophy but also in implant geometry and in the manufacturer-recommended cement used, these results should be interpreted as construct-level effects rather than as evidence that fixation philosophy alone determines fracture resistance.

## Data Availability

The datasets generated and/or analysed during the current study are available from the corresponding author on reasonable request.

## Abbreviations

CMK: Charnley–Marcel–Kerboull
CT: Computed tomography
IQR: Interquartile range
PMMA: Polymethylmethacrylate
PPF: Periprosthetic femoral fracture
THA: Total hip arthroplasty
